# Methodology of Object Reconstruction by Photogrammetry and Structured-Light Scanning for Industrial 3D Visualisation

**DOI:** 10.3390/s25237177

**Published:** 2025-11-24

**Authors:** Anastasiia Nazim, Martin Kondrát, Kamil Zidek, Jan Pitel

**Affiliations:** Faculty of Manufacturing Technologies with the Seat in Prešov, Technical University of Košice, Bayerova 1, 080 01 Prešov, Slovakia; anastasiia.nazim@tuke.sk (A.N.); kamil.zidek@tuke.sk (K.Z.); jan.pitel@tuke.sk (J.P.)

**Keywords:** digital twin, object reconstruction, optical scanning methods, 3D geometric accuracy

## Abstract

In the context of accelerating digitalization, reliable object reconstruction represents a key prerequisite for developing accurate and functional digital twins. This study introduces a unified evaluation methodology designed to assess and compare optical 3D scanning technologies in terms of geometric accuracy, data completeness, and model consistency. The framework integrates all essential stages of digital reconstruction—from data acquisition to quantitative validation—ensuring reproducibility and comparability of results across different optical systems. To verify its applicability, two optical principles, photogrammetry and structured-light scanning, were implemented on the autonomous mobile robot MiR100. The reference CAD model in a 1:1 scale served as the ground-truth geometry for all analyses. Evaluation procedures included visual inspection, dimensional measurements, and statistical error analysis performed in MeshLab, CloudCompare, and MATLAB. The results confirmed that photogrammetry provides high-quality textural detail but suffers from geometric noise and scale drift (relative error > 10%), whereas structured-light scanning delivers more stable and metrically accurate results. In particular, the scanner mode achieved the highest precision, with a mean deviation of 17.4 mm, RMSE of 26.8 mm, and relative error of 7.6%. The proposed methodological framework thus establishes a reproducible basis for evaluating 3D reconstruction accuracy and supports the integration of optimized digital models into digital twin environments.

## 1. Introduction

In the context of modern digitalization, three-dimensional reconstruction technologies are gaining increasing significance due to their ability to accurately reproduce the geometric characteristics of objects in a digital format. The main principle is to capture the geometric parameters, shape, spatial location, and in some cases, the textural characteristics of the surface [1]. These technologies open new opportunities for analysis, preservation, reconstruction, and visualization of objects across various fields of science, industry, and culture. Therefore, research into field techniques for 3D reconstruction is not only relevant but also essential for the further development of interdisciplinary approaches in both science and practice.

3D reconstruction methods provide a high level of accuracy and detail in the digital reproduction of objects. This significantly enhances the efficiency of modeling, quality control, reverse engineering, and prototyping processes. In industrial production, it contributes to cost reduction, shorter product development cycles, and increased competitiveness of enterprises [2].

In the development of digital twins, 3D reconstruction methods provide the essential geometric foundation for accurate virtual representation [3]. By capturing the real-world geometry and texture of an object, scanning technologies, such as structured-light scanning, and techniques, such as photogrammetry, enable precise digital reconstruction that supports further simulation and analysis. This ensures consistency between the physical and digital domains and allows updates of the model when the real object changes, forming the basis for reliable digital twin creation [4,5].

In manufacturing and technical scenarios, 3D reconstruction acts as a tool for rapid verification of an object’s physical state, allowing the digital twin to function not as a static model but as a dynamic system capable of reflecting current changes [6]. Thus, the use of 3D reconstruction is key for creating high-quality, reliable, and adaptive digital twins [7].

Moreover, the development of accessible technologies such as photogrammetry and structured light makes the digitization process more cost-effective and convenient, enabling 3D scanning to be applied not only in high-tech industries but also in everyday practice. Research on the efficiency of various scanning methods allows for substantiating their appropriateness for specific tasks and contributes to improving the quality of digital reconstructions [1].

Within the scope of this study, optical 3D scanning will be used to reproduce the 3D reconstruction of an object, specifically employing two implementation principles: photogrammetry and structured light. Photogrammetry allows the formation of 3D models based on a series of photographs taken from different angles, providing flexibility and accessibility in the 3D reconstruction process. Structured light technology, in turn, ensures high accuracy and completeness of the geometric model through the projection of light patterns and analysis of their distortions.

Combining these approaches within a single study enables a comparative analysis of their efficiency based on accuracy, data completeness, and suitability for integration into a digital twin. This approach provides a foundation for selecting the optimal scanning method for a specific object and for developing recommendations regarding its practical application in industry.

## 2. Related Works

Recent studies highlight that 3D scanning has evolved from a laboratory tool to an integrated tool in Industry 4.0 environments [1,2,3]. Optical scanning methods significantly reduce prototyping costs and accelerate the feedback between design and production, while maintaining operational accuracy in manufacturing systems. However, persistent challenges such as calibration reliability, scale consistency, and environmental sensitivity continue to hinder full standardization [1,2,3]. A comprehensive survey by Daneshmand et al. presents a wide-ranging overview of 3D scanning methodologies, including close-range, aerial, structure-from-motion photogrammetry, terrestrial and airborne laser scanning, and structured-light and phase-comparison techniques, as well as post-processing methods, such as outlier detection and surface fitting [8]. This foundational work establishes that diverse techniques each offer advantages and trade-offs in accuracy, resolution, and applicability. More recently, Bin Cui et al. provided an in-depth overview of both image-based and scanner-based 3D modeling technologies, classifying various 3D scanners by their operating principles and discussing their application domains [9].

### 2.1. Photogrammetry in Digital Twin Reconstruction

Photogrammetry, particularly Structure-from-Motion (SfM) and Multi-View Stereo (MVS), is an economically efficient and widely accessible method for generating dense 3D reconstructions from two-dimensional images. In the context of digital twin development, photogrammetry enables accurate visual and geometric representation of physical objects, industrial systems, and built environments, forming a crucial foundation for virtual replication and analysis. Recent research emphasizes its expanding use in digital twin applications. Modern scanning tools combined with photogrammetric modeling can provide a reliable geometric basis for industrial and architectural systems [10]. Crowdsensed photogrammetry, where data are captured using smartphones or compact cameras, has proven capable of producing metrically accurate 3D models of cultural heritage objects [11]. This approach increases accessibility while maintaining sufficient precision for documentation and visualization tasks. A hybrid approach integrating photogrammetry with laser scanning further improves completeness and alignment, reducing typical issues such as lighting variations, occlusions, and mesh inconsistencies [12]. In addition, AI-assisted algorithms now support real-time image calibration, automatic scaling, and texture enhancement, improving both accuracy and processing efficiency [13,14]. Despite these advantages, traditional photogrammetry still faces limitations when dealing with reflective or low-texture surfaces and depends heavily on environmental lighting. The need for precise scale calibration remains critical [15].

### 2.2. Structured-Light Scanning Techniques

Structured light scanning (SLS) remains highly valued for automation, precision, and affordability. Structured-light scanning is a promising technique for automatic and accurate 3D reconstruction using a consumer camera and video projector setup [16]. Meanwhile, a specific review focusing on online-structured light scanning highlights its high-precision capabilities for small- to medium-sized objects, addressing device- and algorithm-level challenges and avenues for improvement [9]. Recent innovations continue to refine SLS accuracy and speed. A 2025 study introduced an improved scanning-direction calibration technique in line-structured light systems to boost efficiency and precision in industrial measurement contexts [17]. Advanced structured-light methods also tackle challenging surfaces. For specular (highly reflective) surfaces, combining gray-code patterns with phase-shifting techniques achieves dense sub-pixel accuracy reconstructions [18,19]. In related work, Kniaz et al. integrated structured-light scanning with deep learning—specifically a LineMatchGAN model—to refine optical flow and reduce reconstruction artifacts, achieving significant improvements in stereo matching error rates [20].

### 2.3. Applications Toward Digital Twin Visualization

Digital twin visualization necessitates methods capable of generating accurate, textured 3D models. A study in construction site digital twins combined 3D scanning and camera-based reconstruction of both static scenes and dynamic elements to produce near–real-time, high-fidelity models of site activity [21]. Moreover, the concept of a digital twin of a structured-light metrology system introduces a meta-level digital model representing the metrology scanner, enabling enhanced calibration, parameter optimization, and automated control for improved reconstruction quality [22]. Earlier work on data fusion demonstrates combining photogrammetry (SfM), laser scanning, and structured-light scans to enhance textured 3D models—especially for cultural heritage objects—via semi-automatic 2D–3D registration and optimization of color projection.

### 2.4. Analysis of Existing Research

Although numerous investigations have been conducted over the past decade, existing studies remain methodologically fragmented. The literature indicates that the field still lacks a unified procedure for quantitative accuracy evaluation [23,24]. Most research relies on qualitative visual assessment or simplified geometric shapes that do not represent industrial complexity, while scanning parameters and environmental conditions are inconsistently reported [9,19,25]. Comparative analyses typically focus on a single technique—either photogrammetry [13,25,26] or structured-light scanning [17,19,27]—and only a few investigations consider both under identical experimental setups. As a result, direct cross-method comparison and standardized performance indicators are still underdeveloped. To overcome these limitations, the present study proposes a unified methodological framework that combines photogrammetry and structured-light scanning in a shared evaluation environment using MeshLab, CloudCompare, and MATLAB. The framework integrates visual, dimensional, and statistical analyses applied to the same industrial object with a CAD reference model. This approach provides a reproducible, quantitative, and application-oriented foundation for selecting optimal 3D scanning technologies in reverse-engineering and digital-twin manufacturing contexts.

## 3. Overview of 3D Scanning Methods and Tools

**Figure 1:**
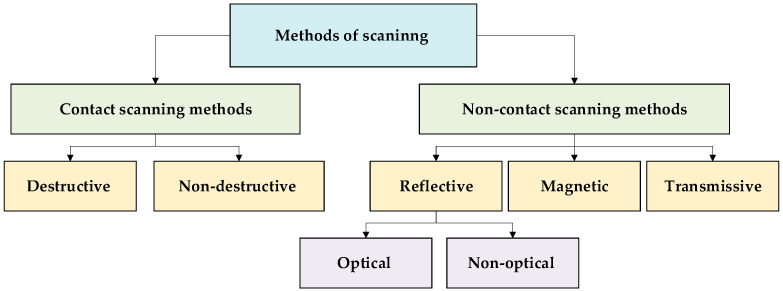
Methods of object scanning.

Depending on the principle of interaction with the object, 3D scanning methods are divided into two fundamental groups: contact—with direct physical contact with the surface; contactless—which perform measurements remotely using light, laser, magnetic, or other fields (see Figure 1). Both types have their own advantages, limitations, and specific uses, which determine the appropriateness of their application in a particular professional field.

(a) Contact scanning methods

Contact methods involve the physical interaction of a measuring device with an object. They are divided into:

- Destructive—used when the object is subject to mechanical processing to reproduce its internal structure. Such methods include milling and turning, which allow you to create a digital model based on layer-by-layer material removal [28].
- Non-destructive—provide geometry fixation without destroying the object. A typical example is the use of a mechanical manipulator or coordinate measuring machine, where the coordinates are read using a contact probe [29].

(b) Non-contact scanning methods

Non-contact methods allow objects to be read without direct physical contact, making them more versatile and safer for a wide range of applications [30]. These methods are classified into:

- Reflective methods are based on the analysis of reflected light. This method includes optical scanning methods, which include photogrammetry and structured light scanning, and non-optical scanning methods, which are based on other types of reflection (such as ultrasound or infrared radiation) [31,32].
- Magnetic methods use a changing magnetic field to reproduce the structure of an object, for example, magnetic probes and magnetic resonance imaging (MRI) [33,34].
- Transmissive methods are based on the passage of radiation through an object. The most common method is computed tomography (CT), which allows the reproduction of both the external and internal structure of objects [35].

### 3.1. Optical Scanning Methods

Optical methods belong to reflective non-contact technologies. The principle of operation of this method is based on the analysis of reflected light radiation to restore the spatial geometry of the object [5]. It is also possible to divide it into two parts: active or passive systems, depending on whether the scanner creates its own radiation or uses external light. The main advantage of optical scanning is high resolution, measurement speed, and the absence of the need for physical contact with the object [1]. Optical methods include photogrammetry, structured light scanning, and laser scanning.

**Figure 2:**
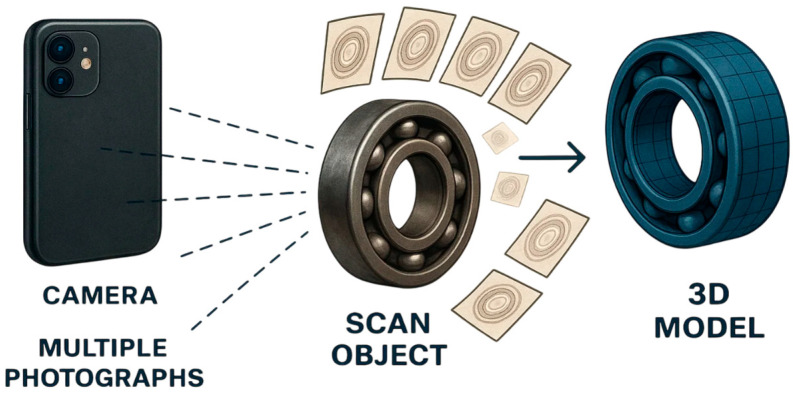
Process of 3D object reconstruction using photogrammetry.

**Photogrammetry** provides reliable information about the three-dimensional position of the points of the object, based on the analysis of a set of images (See Figure 2). Three-dimensional data is presented in the form of a three-dimensional point cloud or a three-dimensional grid.

The use of photogrammetry is becoming widespread in every field. For example, in industrial applications, researchers are studying the practicality of using consumer equipment (including the iPhone 15 Pro with photogrammetry capabilities) to create 3D models that are accurate virtual models of physical objects. They demonstrate that photogrammetry can achieve a measurement accuracy within 5% of the object’s actual dimensions, which is sufficient for most tasks in Industry 4.0 [25]. A fully automated system has also been created that uses photogrammetric images from drones and the Neural Radiance Fields (NeRF) method to create accurate 3D models. The system analyses deviations from the reference model and identifies potential defects in equipment or building structures. Photogrammetry is becoming an integral part of repair processes. The results show that short-range photogrammetry is an effective inspection tool in the repair process chain, focusing on the ability to detect small features and measure gaps with submillimetre dimensions [36].

**Figure 3:**
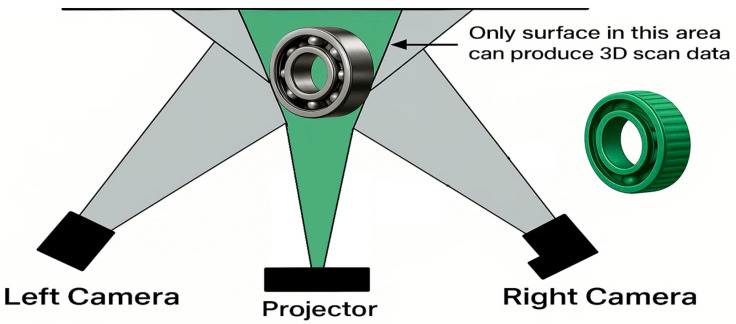
Process of 3D object reconstruction using structured-light scanning.

**Structured light scanning** works by projecting patterned light lines from a fixed source (like a projector), capturing detailed coordinates of the scanned model, including colors and textures (See Figure 3). Structured light scanning is widely used in various fields.

For example, in the study of [27], this scanning technology was compared using different scanners for clinical use of 3D models [37], which presented the first methodological study of the use of SLS for 3D scanning of historical clothing. They developed a step-by-step methodology for scanning museum exhibits (in particular, the costume of the Emir of Bukhara) in situ [38], and compared structured light with coordinate measuring machines (CMM) for the evaluation of 3D-printed parts. It was proven that SLS gives similar results to CMM for the analysis of geometric deviations and can be used in both post-process (offline) and process (in-process) control. A new neural network was proposed for fast phase analysis in projection profilometry. It replaces the classical multi-step phase shift methods, reducing the reconstruction time to 16.7% of the traditional one without losing accuracy (≈40 μm). This opens new possibilities for fast and accurate scanning of objects with high dynamic variability of reflectivity.

### 3.2. Software Tools for Editing and Comparing Scanned Models

There is now a lot of software on the market, both commercial and open source. With the help of this software, you can improve the quality of the model, fix imperfections, and prepare the model for further use. Open-source software includes MeshLab (2023.12) and CloudCompare (2.13). Commercial software includes ZEISS Reverse engineering (2.8), Geomagic Design X (2024.2), Zbrush (2024), and Recreate (Hexagon) (2023.4).

Each of the examined software tools shares several common features. They support key algorithms for processing scanned models, including scan alignment (particularly through the ICP—Iterative Closest Point—algorithm), noise filtering and anomaly removal, as well as surface generation from point clouds or meshes (for example, using Poisson Reconstruction, Radial Basis Functions (RBF), or Non-Uniform Rational B-Splines (NURBS)). All the programs also offer functionalities such as geometry editing (e.g., stitching, hole filling, decomposition, and topological reconstruction); analysis of deviations from a reference model or between different scans; and generation of a digital 3D model ready for export or further processing.

These tools are commonly used at the same stage of the workflow—immediately after the scanning process. However, their specific use cases and intended applications vary. MeshLab is identified as a 3D mesh editor optimized for working with triangular point clouds and polygonal models. It provides tools for cleaning, alignment, remeshing, polygon reduction, hole filling, and texturing. CloudCompare focuses on point cloud analysis rather than solely working with meshes. It is widely utilized in surveying, architectural documentation, and landscape scanning due to its robust measurement and comparison capabilities [39,40]. ZEISS Reverse Engineering is a commercial software product that supports certified metrological analysis and integration with high-precision measurement hardware. It is used for surface reconstruction and for comparing scanned data with reference geometries, especially in industrial quality control [41]. Geomagic Design X serves as a hybrid solution that combines polygon mesh processing with parametric CAD modeling. It acts as a bridge between 3D scanning and CAD platforms such as SolidWorks (2025) and Siemens NX, enabling seamless transition from scanned data to editable CAD models. ZBrush is predominantly applied in the fields of visual effects (VFX), game development, and digital art. Its primary strength lies in artistic modeling, providing a comprehensive set of sculpting tools for working with highly detailed organic forms. Recreate by Hexagon is a dedicated reverse engineering solution used for converting scanned geometry into CAD-ready models suitable for manufacturing workflows. It emphasizes automation and precision in engineering reconstruction.

Finally, MATLAB (R2025a) is increasingly employed for in-depth analysis and comparison of 3D models. It allows users to perform complex mathematical calculations [42], develop custom algorithms for comparing meshes or point clouds, and visualize results through plots, heat maps, and animations. This makes it particularly valuable for scientific and engineering research where numerical precision and algorithmic control are essential.

### 3.3. Accuracy Metrics for Comparing Scanned Models to Their Reference Model

Accuracy metrics allow us to quantify the degree of correspondence between a scanned 3D model and a reference CAD model. The main indicators are the average (absolute) deviation, the maximum deviation, and the relative error, which reflect the overall level of geometric discrepancies. For more in-depth analysis, the Root Mean Square Error (RMSE), Hausdorff Distance, and Normal Deviation are used. These metrics are key for quality verification, defective detection, and improvement of digital object copying technologies.

**Relative error equation:**

(1) $$\varepsilon =(\frac{∆X}{Xmax})\times 100\%\;$$

∆X—the absolute error.

Xmax—the maximum value (e.g., the maximum deviation between models).

ε—the relative error in percent.

**Root Mean Square Error (RMSE):**

It estimates the mean square deviation between corresponding points of two models.

(2) $$RMSE=\sqrt{\frac{1}{N}\sum_{i-1}^{N}{∥p_{i}-q_{i}∥}^{2}}$$

*p_i_*—coordinates of the point on the scanned model.

*q_i_*—coordinates of the corresponding point on the reference model.

*N*—total number of point pairs being compared.

∥*p_i_* − *q_i_*∥—Euclidean distance between the two points.

**Hausdorff Distance:**

It determines the largest deviation between two sets of points.

(3) $$d_{H}\left(P,Q\right)=max\;\left\{\underset{p\in \mathrm{P q}\in Q}{\sup\inf}∥p-q∥,\;\underset{q\in \mathrm{Q p}\in P}{\sup\inf}∥q-p∥\;\right\}$$

*P*—set of points from the scanned model.

*Q*—set of points from the reference model.

*inf*—the minimum distance to the closest point.

sup—the maximum of those minimum distances.

∥p−q∥—Euclidean distance between points p and q.

## 4. Methodology and Experimental Setup

### 4.1. General Framework

**Figure 4:**
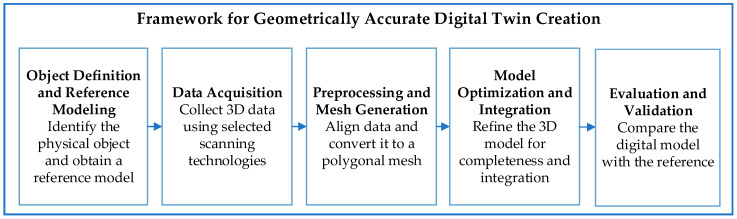
General methodology for the creation and validation of a digital twin using optical 3D scanning methods.

The proposed methodological framework defines a standardized process for creating geometrically accurate digital twins through optical 3D scanning. It includes five consecutive stages that ensure reproducibility and comparability of results across different technologies (Figure 4).

1. Object definition and reference modeling—Select a physical object and define a precise reference model (CAD or high-accuracy scan) as ground truth.
2. Data acquisition—Capture 3D data using optical methods such as photogrammetry or structured-light scanning, defining parameters like distance, overlap, lighting, and point density.
3. Pre-processing and mesh generation—Align captured datasets, remove noise, and reconstruct the surface mesh using algorithms such as ICP or Poisson reconstruction.
4. Model optimization and integration—Refine the model to ensure watertight topology, repair discontinuities, and reduce redundant polygons for efficient visualization and analysis.
5. Evaluation and validation—Compare the reconstructed model with the reference using quantitative metrics (mean deviation, RMSE, Hausdorff distance) and qualitative visual assessments.

This framework provides a reproducible methodological basis for assessing 3D scanning technologies and integrating resulting models into digital twin environments across industrial and research contexts.

### 4.2. Implementation of the Methodology

To verify the proposed framework, the methodology was implemented on the MiR100 autonomous mobile robot (Mobile Industrial Robots ApS, Odense, Denmark) as a representative case study. The implementation comprised the use of scanning technologies and software, object selection, a detailed scanning workflow, and a summary of key parameters.

#### 4.2.1. Scanning Technologies and Software

**Figure 5:**
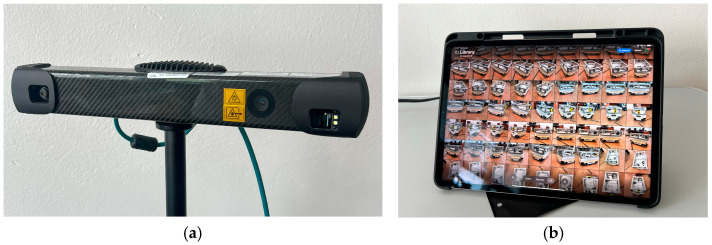
Chosen devices for scanning: Photoneo MotionCam-3D Color M+ (**a**); iPad Pro 11 (**b**).

Two complementary optical 3D scanning methods were applied to enable comparative analysis:

- Photogrammetry—image acquisition was performed using an Apple iPad Pro 11 (M4 chip) (Apple Inc., Cupertino, CA, USA) equipped with a 12 MP wide-angle camera (See Figure 5b). The device was used only for photographic capture; its built-in LiDAR sensor was not employed. A total of 128 overlapping images (≈70–80% overlap) were taken under uniform ambient lighting from multiple viewpoints. The photos were exported to RealityCapture (Epic Games), where reconstruction was performed using Structure-from-Motion (SfM) and Multi-View Stereo (MVS) algorithms to generate a textured mesh (OBJ format).
- Structured light scanning—conducted using a Photoneo MotionCam-3D Color M+ (Photoneo s.r.o., Bratislava, Slovakia) operating in scanner and camera modes (See Figure 5a). The system provides a 1680 × 1200 px spatial resolution, an optimal working distance of 0.9 m, and an accuracy below 0.3 mm. Data acquisition and meshing were performed using PhoXi Control (1.14.0) and PhoXi Instance Meshing software (2.0.0).

All resulting meshes were post-processed in MeshLab, aligned with the CAD reference model in CloudCompare, and statistically analyzed in MATLAB.

#### 4.2.2. Selection of the Object for Scanning

**Figure 6:**
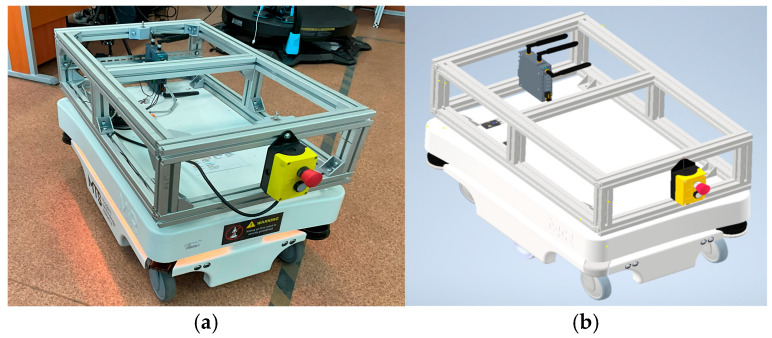
The autonomous mobile robot MiR100 physical prototype (**a**) and its corresponding CAD model (**b**).

The MiR100 mobile robot was selected as the experimental object due to its medium size, moderate geometric complexity, and variety of materials (metal, plastic, glass) (See Figure 6a). The object combines planar and curved surfaces, making it suitable for testing scanning accuracy and data completeness [43].

A detailed 1:1 CAD model of the robot was available and served as the ground-truth reference for all quantitative evaluations, ensuring reproducibility and metric consistency (See Figure 6b).

#### 4.2.3. Scanning and Model Generation Process

Both scanning methods followed the unified workflow described in Section 4.1, with specific adjustments to account for the characteristics of each technology.

**Figure 7:**
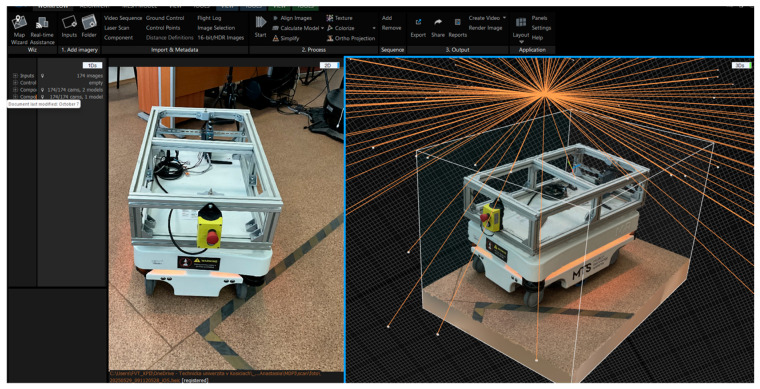
Used software for photogrammetry: RealityCapture.

**Figure 8:**
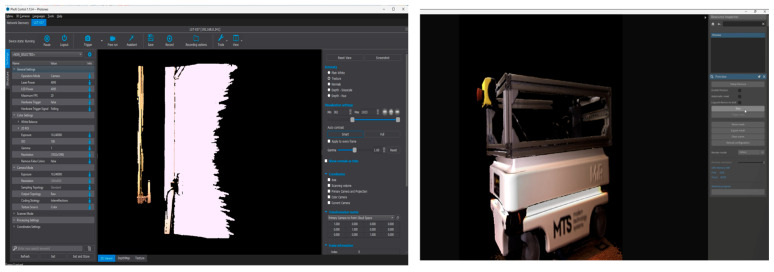
Used software for structural light scanning: PhoXi Control and PhoXi Instance Meshing.

1. The preparation and environment setup were conducted under controlled conditions. The MiR100 robot was positioned on a matte, non-reflective surface to minimize glare and reflections, while evenly distributed ambient lighting was used to avoid shadows and maintain uniform image exposure throughout the scanning process.
2. During photogrammetric acquisition, a total of 174 photographs of the MiR100 robot were captured under uniform lighting conditions, following a circular trajectory at a distance of ≈1.1 m with 70–80% overlap (Figure 7). This configuration yielded an average Ground Sampling Distance (GSD) of ≈1.2 mm/pixel, ensuring sufficient geometric detail. All images were processed in RealityCapture (1.4.2) (Epic Games, Cary, NC, USA), where automatic alignment, dense reconstruction, and mesh generation were performed without external scale markers or control points. The reconstruction relied solely on intrinsic camera parameters and the built-in photogrammetric workflow. High-detail and sharp-geometry settings were applied, resulting in a textured 3D mesh of about 5.2 million polygons. Figure 8 shows the RealityCapture interface with the aligned photographs and the reconstructed model of the MiR100 robot.

For the structured-light scanning, data were collected in both scanner and camera modes. In scanner mode, 3D frames were recorded at 2 fps to achieve high precision, whereas in camera mode, the frame rate was increased up to 20 fps to allow faster data capture and broader coverage of the object’s geometry. Figure 8 shows the user interface of the PhoXi Control and PhoXi Instance Meshing software, illustrating the configuration of scanning parameters and the resulting 3D data visualization.

**Figure 9:**
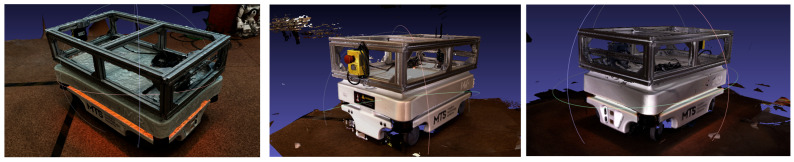
Three-dimensional models without optimization and noise filtering.

**Figure 10:**
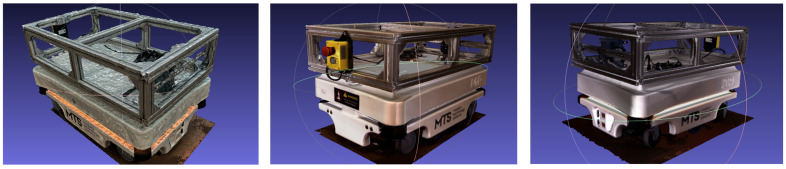
Mesh optimization and noise filtering in MeshLab.

3. After data acquisition, all datasets were registered in a unified coordinate system to maintain consistent orientation relative to the reference CAD model. Importantly, no additional scaling or geometric transformations were applied to the reconstructed models after processing. Each dataset was analyzed in its original scale to preserve the inherent dimensional characteristics of the respective scanning technology, ensuring objective comparison between photogrammetry and structured-light results.
4. The mesh reconstruction and texturing (Figure 9) were performed separately for each method. In the photogrammetric workflow, the image dataset was processed in RealityCapture, where the Structure-from-Motion (SfM) and Multi-View Stereo (MVS) algorithms were used to generate a polygonal mesh with high-resolution photo-based textures. In contrast, the structured-light data were processed in PhoXi Instance Meshing, which directly converted the point clouds into dense color meshes without external texturing.

5. Subsequently, both models underwent post-processing and optimization in MeshLab (See Figure 10). This included noise filtering, mesh repair, and polygon reduction to improve surface smoothness while maintaining geometric fidelity. The cleaned and optimized meshes were then imported into CloudCompare, where they were aligned with the reference CAD model to prepare for further evaluation.

6. Finally, both models were validated against the reference CAD geometry to confirm surface completeness and dimensional consistency. The detailed quantitative and qualitative evaluation of these models is presented in Section 5.

## 5. Evaluation of 3D Reconstruction Methods

This section presents the results of the 3D reconstruction and evaluation process. The analysis is divided into two complementary parts: qualitative analysis, focused on visual completeness and surface quality of the reconstructed models, and quantitative analysis, which assesses dimensional accuracy and statistical deviations relative to the reference CAD geometry.

### 5.1. Qualitative Analysis of 3D Reconstruction

During the visual comparison of the three applied 3D scanning methods—photogrammetry, structured light in camera mode, and structured light in scanner mode—distinct differences in visual quality were observed.

**Figure 11:**
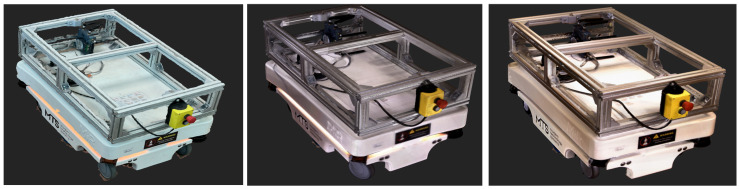
Textured 3D models of the MiR100 mobile robot: photogrammetry (**left**), structured light in camera mode (**middle**), and structured light in scanner mode (**right**).

**Figure 12:**
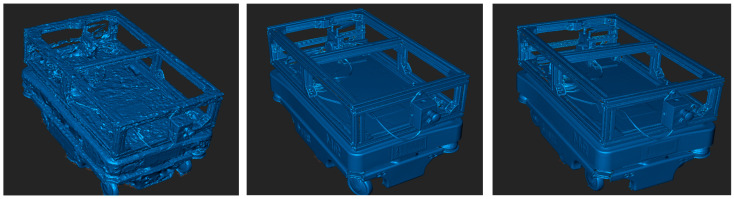
Mesh representations of the MiR100 robot: photogrammetry (**left**), structured light in camera mode (**middle**), and structured light in scanner mode (**right**).

Figure 11 and Figure 12 illustrate the reconstructed models of the MiR100 robot. The photogrammetric model provides realistic textures and color fidelity due to the use of photographic data; however, its surface mesh exhibits noticeable noise, and some fine details appear blurred or distorted.

The structured-light model in camera mode shows clearer geometry and more stable planar surfaces while maintaining sufficient visual detail. The structured-light model in scanner mode demonstrates the smoothest surface and the most defined contours, with minimal visible artifacts.

### 5.2. Quantitative Analysis of 3D Reconstruction Accuracy

This section presents a quantitative comparison of the reconstructed 3D models with the reference CAD geometry. The assessment focuses on measuring dimensional deviations, point-to-surface distances, and statistical accuracy indicators to verify the geometric consistency of each scanning method.

#### 5.2.1. MeshLab Measurements

To evaluate the geometric accuracy of the reconstructed models, MeshLab was used to align the CAD reference model of the MiR100 robot with the 3D models obtained from Reality Capture and structured-light scanning. The alignment was performed manually by adjusting the models in a unified coordinate system, followed by dimensional verification using the built-in ruler tool.

**Figure 13:**
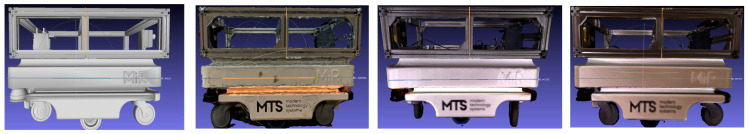
Side views of the MiR100 mobile robot showing the measured length and height dimensions.

**Figure 14:**
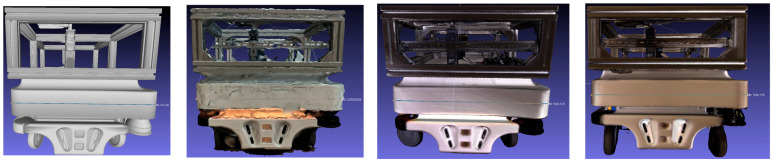
Front views of the MiR100 mobile robot showing the measured width dimensions.

Figure 13 and Figure 14 show the measurement configuration used for assessing the robot’s principal dimensions: length (L) and height (H) from the side view, and width (W) from the front view. Dimensional verification was performed using distinct, repeatable geometric features on the robot, including the lower chassis edge, upper frame corners, and wheel centers, which are clearly visible in both the CAD and reconstructed models. These features served as virtual reference points for consistent measurement alignment. The identification of these reference elements allowed all dimensional measurements to be taken between the same geometric locations in each model, ensuring comparability without the need for physical markers or targets.

The dimensional comparison summarized in Table 1 shows that the structured-light models exhibit minimal deviations from the CAD reference—below 1% in length and width, and up to 10% in height due to limited visibility of upper structural elements. In contrast, the photogrammetric model shows major inconsistencies exceeding 70%, indicating a loss of scale during reconstruction.

#### 5.2.2. Accuracy Evaluation Using CloudCompare

The geometric accuracy of the reconstructed models was evaluated in CloudCompare using the Cloud-to-Mesh (C2M) distance computation.

The photogrammetric model generated in RealityCapture was included in the previous dimensional analysis (Section 5.2.1), where significant scale discrepancies were identified relative to the CAD reference. Because the model did not preserve the absolute 1:1 scale of the physical object, it was excluded from quantitative C2M evaluation in CloudCompare. Although manual rescaling could potentially adjust the model dimensions, it would artificially influence the deviation values and compromise the objectivity of the results. Consequently, only the structured-light datasets were used for quantitative accuracy assessment.

**Figure 15:**
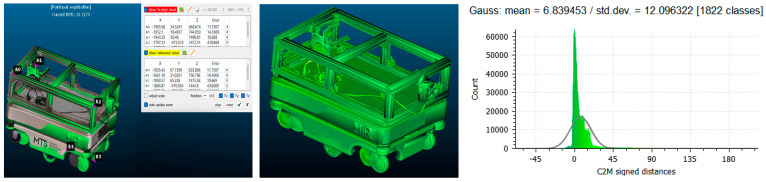
Comparison of the CAD model and the scanned model obtained using structured light scanning in scanner mode in CloudCompare.

**Figure 16:**
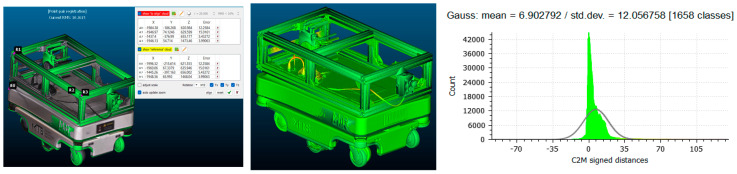
Comparison of the CAD model and the scanned model obtained using structured light scanning in camera mode in CloudCompare.

Each scanned model was aligned to the reference CAD geometry using the iterative closest point (ICP) algorithm, guided by manually defined control points placed on repeatable geometric features such as frame corners and wheel hubs (See Figure 15 and Figure 16 (left)).

The C2M analysis calculated signed point-to-surface distances for all mesh vertices within a ±45 mm range, chosen to capture the full extent of local deviations. The default CloudCompare palette was applied, where green marks near-zero deviation (≤±1 mm), yellow and red represent positive offsets, and blue corresponds to negative deviations (See Figure 15 and Figure 16 (middle)). The dominance of green areas confirms high geometric conformity between the scanned and reference models.

For the structured-light scanner mode, the alignment achieved a mean deviation of 6.84 mm, an RMS error of 11.21 mm, and a standard deviation of 12.10 mm. For the camera mode, the RMS error was 10.26 mm, with a mean deviation of 6.90 mm and standard deviation of 12.06 mm. The deviation histograms (See Figure 15 and Figure 16 (right)) show a sharp central peak within ±10 mm, indicating that most surface points closely match the CAD reference. A slight rightward skew suggests a minor mesh expansion, likely due to optical overestimation during reconstruction.

#### 5.2.3. Analysis in MATLAB

All quantitative analyses were performed in the MATLAB R2025a environment using a custom script developed for automatic processing, alignment, and numerical evaluation of the scanned models. The workflow consisted of four steps:

(1) model import and centering,
(2) point cloud surface generation,
(3) spatial registration using the ICP algorithm, and
(4) computation and visualization of deviations.

**Figure 17:**
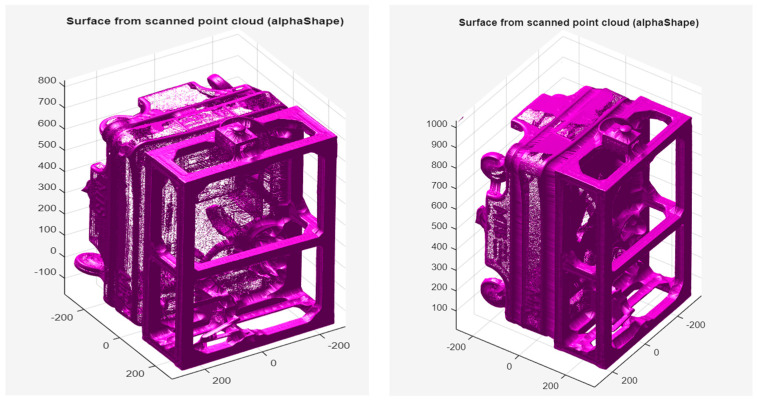
Point clouds of the scanned model generated using the alphaShape function: created with camera mode (**left**); created with scanner mode (**right**).

The CAD model and the scanned models were imported into MATLAB using the stlread() function. The alphaShape algorithm was then applied to reconstruct the outer geometry of each object with the parameter α = 20, generating continuous point clouds that accurately represent the external contours and characteristic features such as the frame, wheels, and protrusions (See Figure 17. Because alphaShape encloses the point set with a sphere of a defined radius, it reproduces only the external shell, while the inner cavities remain unfilled; smaller α values could capture finer internal details but would increase noise. Figure 17 compares the scanned models: the camera-mode model (left) shows visible gaps and a less dense structure due to limited viewing angles, whereas the scanner-mode model (right) exhibits a denser, more uniform surface, confirming higher scan quality and data completeness.

**Figure 18:**
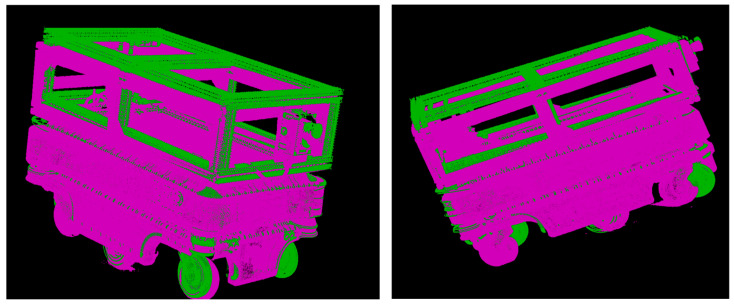
Visualization of spatial alignment of the CAD model (green) with the scanned model (pink), created with camera mode (**left**) and with scanner mode (**right**).

Subsequently, both scanned models were aligned with the reference CAD model in MATLAB using the pcregistericp() function, which implements the Iterative Closest Point (ICP) algorithm (See Figure 18). This automatic registration procedure ensured precise geometric alignment between the datasets. In the visualization, the CAD model is displayed in green, while the scanned data are shown in pink—on the left for the camera mode and on the right for the scanner mode. The resulting overlap demonstrates accurate spatial correspondence between the reference and scanned geometries.

**Figure 19:**
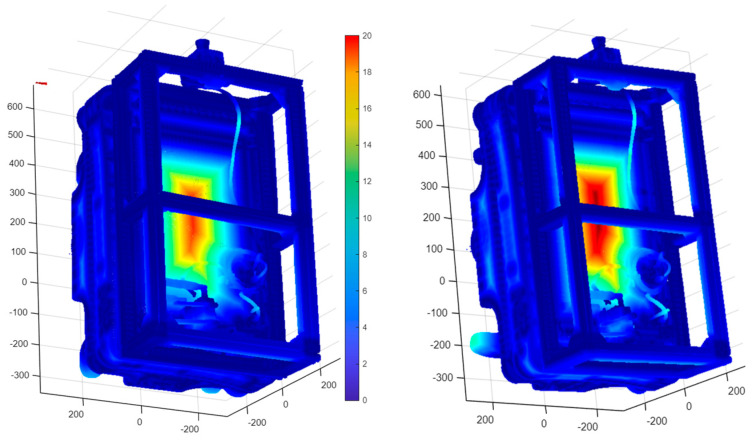
Heat map of deviations: created with camera mode (**right**); created with scanner mode (**left**).

To quantitatively analyze the deviations between the corresponding points of the two models, a heat map was constructed. Images display the magnitude of the deviations using a color scale: from blue (minimum errors) to red (maximum). The analysis showed that most of the surface has small deviations, while the largest ones are observed in individual areas of complex internal geometry, which are probably less accessible for scanning (See Figure 19).

For quantitative evaluation, graphical visualizations were created that display the distribution of point-to-surface deviations between CAD models and scanned models. The graphs include deviation histograms and spatial maps that show the correlation of point coordinates with their deviation values, allowing the identification of areas with higher geometric variance.

- Deviation Histogram

**Figure 20:**
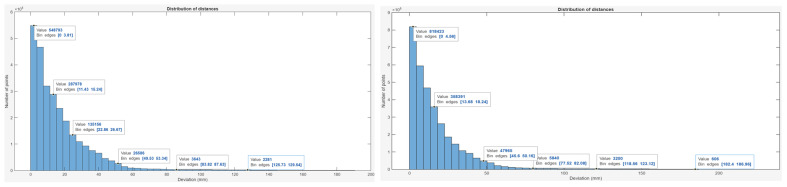
Deviation histogram of scanned model using camera (**left**) and scanner (**right**) mode.

Figure 20 shows the histogram of distance distribution between the corresponding points of the scanned and CAD models for both scanning modes.

In the camera mode, the majority of points—548,793 (≈20.0% of the total 2,747,673)—fall within the 0–3.81 mm deviation range, indicating high local reconstruction accuracy. Another 287,978 points (≈10.5%) lie within 11.45–15.24 mm, and 135,156 points (≈4.9%) are within 22.96–26.67 mm. Only 2281 points (≈0.08%) exceed 120 mm, showing that large deviations occur rarely and have minimal statistical impact.

In the scanner mode, the histogram shows that 818,423 points (≈24.7% of the total 3,317,064) have deviations within 0–4.56 mm, confirming precise surface correspondence with the CAD reference. An additional 358,391 points (≈10.8%) lie in the range of 13.68–18.24 mm, while around 8000 points (≈0.24%) exceed 100 mm, with the maximum deviation reaching 226.5 mm.

- Deviation Color Map

The deviation maps illustrate the correlation between the point coordinates (X, Y) and the measured deviations (Z) between the scanned and CAD models, expressed in millimeters (mm). The color scale represents the magnitude of deviation, where blue indicates minimal differences (below 20 mm) and red marks local peaks of higher deviations.

**Figure 21:**
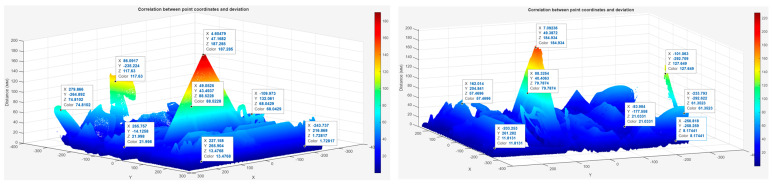
Deviation color map of scanned model using camera (**left**) and scanner mode (**right**).

In the camera mode model (Figure 21 left), the deviation values range from 1.7 mm to 187.3 mm, with the highest peaks located at the upper frame elements (coordinates X ≈ 4.6 mm, Y ≈ 47 mm). Most of the surface remains within the 0–80 mm range, indicating generally uniform geometry. In the scanner-mode model (Figure 21, right), deviations range from 8.1 mm to 184.9 mm. The peak deviation (≈185 mm) is also observed in the upper frame, but the overall distribution is smoother and more homogeneous across the structure, with the majority of points below 60 mm.

- Graph of distances for each point

**Figure 22:**
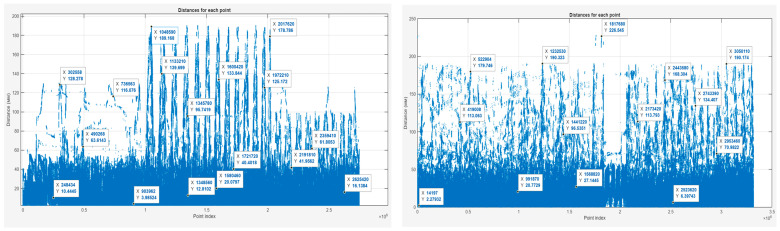
Graph of distances for each point of the scanned model using the camera mode (**left**) and the scanner mode (**right**).

Figure 22 shows the distance plots representing deviations between corresponding points of the scanned and CAD models as a function of the point index.

In the camera-mode model (left), a dense cluster of points is concentrated within deviations below 40 mm, indicating high local consistency. Several distinct peaks exceeding 150 mm are visible, corresponding to individual alignment differences or local reconstruction artifacts.

In the scanner-mode model (right), recurring peaks appear at approximately 113 mm, 134 mm, 168 mm, 190 mm, and above 220 mm, which reflect repeated structural deviations along the upper elements of the frame.

#### 5.2.4. Statistical Precision and Point-Based Computation

All quantitative accuracy metrics in Table 2 were computed automatically using a MATLAB script based on the KD-tree nearest neighbor method.

According to the final numerical metrics, the average deviation between the scanned and reference models was 17.70 mm for the camera-modal and 17.39 mm for the scanner mode. This indicates an almost identical average accuracy of both approaches. At the same time, the maximum deviation was significantly larger in the scanner mode—227.83 mm versus 190.31 mm—which is confirmed by the corresponding visualizations of local peaks on the distance graphs. The standard deviation also slightly exceeds the indicator for the scanner (20.38 mm) compared to the camera (20.23 mm), which indicates a slightly larger spread of values. At the same time, the relative error was higher in the camera-mode model—9.30% versus 7.63%—which indicates the superiority of the scanner approach in the context of overall consistency with the CAD model. The root mean square error (RMSE) indicator practically does not differ between the two methods: 26.88 mm (camera) and 26.8 mm (scanner). This confirms that both methods demonstrate similar efficiency in the overall reconstruction of the object. The most significant difference was recorded for the Hausdorff distance metric, which was 190.31 mm for the camera mode and 227.83 mm for the scanner mode, indicating the existence of a larger maximum spatial discrepancy in the scanner model. Graphical analysis of the distance distribution showed that in both cases, the majority of points—over 500,000, corresponding to approximately 92.6% of all measured points—have deviations within 20 mm, while the number of points with significant deviations (over 100 mm) remains extremely low (<0.1%).

### 5.3. Summary of Evaluation Results

The comparison indicates that photogrammetry achieves realistic textures but lacks geometric reliability; structured light in camera mode offers a balanced compromise between visual detail and accuracy; and structured light in scanner mode delivers the highest overall fidelity, making it most suitable for precision-demanding digital twin applications (See Table 3).

## 6. Discussion of Results

The obtained results confirm that the accuracy and completeness of 3D reconstruction are closely influenced by both the scanning principle and the environmental conditions during data acquisition.

During the scanning process, several sources of error can influence geometric accuracy. In structured-light scanning, calibration drift may occur due to misalignment between the projector and camera axes or temperature-induced shifts in the optical system. These errors were minimized by performing factory calibration of the Photoneo MotionCam-3D system prior to data acquisition and by maintaining stable environmental conditions (constant temperature, uniform lighting, and fixed scanner position). In photogrammetry, geometric noise and scale inconsistencies mainly arise from imperfect camera calibration, variable lighting, and insufficient overlap between images. These effects were mitigated by using a constant circular trajectory, uniform ambient lighting, and high image overlap (≈70–80%). Additional filtering and mesh repair were applied in MeshLab to remove residual surface noise and fill minor holes.

Structured-light scanning demonstrated superior geometric stability compared to photogrammetry, in agreement with previous findings [9,17,19].

This improvement can be attributed to the use of active illumination, which projects a defined light pattern onto the surface. By actively controlling the lighting, the system reduces dependency on ambient conditions, suppresses scale drift, and enhances point-to-point correspondence during surface reconstruction. In contrast, photogrammetry relies on passive image capture, where lighting variation, reflections, and limited texture features can introduce positional errors and cause local geometric distortion.

The scanner mode of the structured-light system achieved the highest dimensional consistency due to its higher frame precision, denser sampling, and lower temporal noise, which together improve spatial registration accuracy. The camera mode, while slightly less precise, offers faster acquisition and broader coverage, which makes it suitable for real-time inspection or rapid prototyping scenarios where absolute metric accuracy is not critical. These observations highlight the inherent trade-off between scanning speed and precision that must be considered when selecting a technology for industrial applications.

Photogrammetry, although capable of producing visually realistic textures, exhibited the largest variation in scale and deviation distribution. This limitation arises from the absence of external control points or physical scale markers, meaning that model reconstruction depends solely on intrinsic camera calibration and estimated focal parameters. The use of reference markers or hybrid setups combining photogrammetry with active optical scanning could therefore significantly improve metric fidelity [10,12,23].

The integrated workflow developed in this study enabled reproducible, software-agnostic comparison across different scanning techniques. The combination of MeshLab, CloudCompare, and MATLAB allowed detailed quantitative and qualitative evaluation, providing both visual and numerical validation of model accuracy. This approach supports standardized assessment of scanning systems and contributes to the methodological foundation for digital twin creation.

Since the 3D scanning process itself does not directly create a model suitable for digital twin visualization. A specific methodology is required to define the individual steps of optimization, alignment, and filtering of the point cloud data obtained during the scanning process. Such a methodological framework provides geometrically consistent models that can be integrated into digital twin environments. The precise geometric dimensions of scanned objects are particularly important for mobile, industrial, collaborative, and humanoid robots or manipulators, where accurate digital twins allow for real-time collision prediction and prevent physical damage to real devices by pre-simulating potential interactions in a virtual environment.

Several limitations of this study should be acknowledged.

- First, the evaluation was performed on a single object (a MiR100 mobile robot), which limits the generalizability of the results to other geometries, materials, and surface treatments.
- Second, the study focused on two optical principles—photogrammetry and structured light—while laser or hybrid systems were not included.
- Third, all experiments were performed in controlled laboratory conditions; variations in ambient lighting, texture, or vibration may affect the accuracy and stability of the scans.
- Fourth, the photogrammetric model was not scaled using external physical markers, which prevented direct quantitative comparison in CloudCompare.

Finally, the MATLAB-based evaluation focused on point-to-point deviation analysis; future work should include curvature-based error estimation and volumetric estimation for a more comprehensive assessment of geometric accuracy.

Future research will concentrate on the integration of next-generation optical and hybrid 3D scanners that provide higher accuracy, faster data acquisition, and enhanced adaptability to complex geometries. Further investigations will address the variability of measurement accuracy across small, medium, and large objects with diverse surface materials and geometric complexity. In addition, the use of expanded datasets will enable automated calibration procedures and cross-validation between different scanning techniques, thereby improving the robustness and reliability of digital-twin generation for industrial applications.

## 7. Conclusions

This paper proposes a unified framework for evaluating optical 3D scanning technologies used in industrial component reconstruction for digital twin integration. The methodology combined structured light and photogrammetric approaches, allowing for an objective evaluation using a standardized workflow implemented in MeshLab, CloudCompare, and MATLAB.

Structured light scanning in scanner mode demonstrated the highest overall geometric accuracy and surface completeness. The lowest relative error (7.63%) and the most stable point cloud density were achieved. Structured light scanning in camera mode provided comparable performance in terms of root mean square error (RMSE) (26.88 mm) and mean deviation (17.70 mm), with slightly reduced accuracy in the vertical dimension due to partial data loss in closed areas. Thus, the camera mode provided a balanced performance between accuracy and data collection efficiency. Photogrammetry, despite producing photorealistic textures, has proven to be less reliable for geometric accuracy due to scale inconsistencies and limited surface completeness.

The developed workflow ensures methodological transparency and adaptability to various optical systems and object types. It serves as a practical tool for standardized assessment of scanning quality in reverse engineering, dimensional inspection, and digital-twin-oriented manufacturing environments.

## Figures and Tables

**Table 1:** Dimensional comparison of reconstructed models with CAD reference.

Digitalization Technology	L ^1^ [mm]	Δ ^4^ L [mm]	W ^2^ [mm]	ΔW [mm]	H ^3^ [mm]	ΔH [mm]
CAD model	848.63	-	543.45	-	239.34	-
Reality Capture model	229.79	618.84	153.93	389.52	62.79	176.55
Structured light in camera mode model	847.55	1.08	540.71	2.74	218.62	20.72
Structured light in scanner mode model	845.97	2.66	539.71	3.74	215.79	23.55

^1^ L—length of the mobile robot. ^2^ W—width of the mobile robot. ^3^ H—height of the structure above the robot. ^4^ **Δ**—deviation from the CAD reference (CAD − Model).

**Table 2:** Accuracy metrics.

Mathematical Method	Camera Mode	Scanner Mode
Mean deviation [mm]	17.70	17.39
Max deviation [mm]	190.31	227.83
Standard deviation [mm]	20.23	20.38
Relative error [%]	9.30	7.63
RMSE [mm]	26.88	26.79
Hausdorff Distance [mm]	190.31	227.83

**Table 3:** Summary of Comparative Results.

Method	Mean Deviation [mm]	RMSE [mm]	Hausdorff Distance [mm]	Relative Error [%]	Optimal Application
Photogrammetry (RealityCapture)	Not comparable (scale mismatch)	–	–	–	Visualization, documentation
Structured light—Camera mode	17.70	26.88	190.31	9.30	General engineering, prototyping, and inspection
Structured light—Scanner mode	17.39	26.79	227.83	7.63	High-precision tasks, CAD integration, quality control

## Data Availability

The original contributions presented in the study are included in the article. Further inquiries can be directed to the corresponding author.
